# Polygenic scores in the study of gene–environment interplay and stress-related psychopathology

**DOI:** 10.3389/fpsyt.2026.1736828

**Published:** 2026-03-05

**Authors:** Mehves Kouris, Robert Kumsta

**Affiliations:** 1 Department of Behavioural and Cognitive Sciences, Faculty of Humanities, Education and Social Sciences, Esch-sur-Alzette, Luxembourg; 2 Laboratory for Stress and Gene-Environment Interplay, University of Luxembourg, Esch-sur-Alzette, Luxembourg; 3 German Center for Mental Health (DZPG), Bochum, Germany

**Keywords:** depression, gene–environment interaction, genome-wide interaction studies, GWIS, GxE, PGS, polygenic scores, SLEs

## Abstract

Polygenic scores (PGS) have become valuable tools for quantifying genetic liability to complex mental disorders. This review discusses the application of PGS in gene–environment interaction (GxE) research, focusing on depression and stress-related phenotypes. We synthesize evidence from studies testing whether genetic risk, as indexed by PGS, moderates the impact of environmental exposures, particularly stressful life events (SLEs), on psychological outcomes. Findings from large-scale population studies lend partial support to the diathesis–stress framework, with significant GxE effects observed between polygenic risk for depression and various stressors. Individuals with higher genetic liability tend to be more vulnerable to the adverse effects of stress, though effect sizes are typically modest. Some studies report robust interactions, whereas others find independent main effects of genes and environment without meaningful moderation. We further review genome-wide interaction studies (GWIS) aiming to identify variants underlying stress sensitivity and emerging research employing PGS to predict psychotherapy response. Despite methodological advances, the limited variance explained by current PGS constrains their immediate translational value. Overall, we outline both the promise and current limitations of PGS-based GxE approaches and highlight opportunities for improving their utility in elucidating how genetic predisposition and environmental adversity jointly shape risk for mental disorders.

## 1 Introduction

With few exceptions, there are no known causes for mental disorders. It is generally accepted that the etiology of mental health problems is multifactorial, with many risk factors interacting across development in a complex way (1). Classical diathesis–stress models conceptualize psychopathology as the result of an interaction between latent vulnerability and environmental stressors, whereby stress activates an underlying predisposition (2). On the side of the psychosocial environment, there are many known risk factors (or stressors) which significantly increases disorder liability, and for many, the effects are long-lasting, contributing to different mental and physical health outcomes. For instance, experiences of adversity, deprivation, or traumatic stress in childhood, but also chronic or traumatic stressors in adulthood pose significant risk for the onset and maintenance of mental health problems (3). Subsequent refinements of the diathesis-stress framework emphasized that both the nature of stressors and their measurement critically determine whether interactions can be detected. (2).

Although is generally accepted that stress in its various forms is a transdiagnostic risk factor, there is considerable heterogeneity in how people respond to such environmental or psychosocial risks, which *gene by environment* interaction findings attribute to interindividual differences at the DNA sequence level.

Initially, the focus of gene-environment interaction research was on candidate genes, where common variants of single genes were tested as moderators between risk factors and mental health outcomes. The most well-known example is the reported interaction between a common variant in the serotonin transporter gene promoter and serious or traumatic life events in relation to depression or depression-related outcomes. Whereas the initial findings by Caspi et al. (4) where replicated by many, the consensus now is that focusing on single variants is questionable for uncovering main or interaction effects (5). It has become clear that single DNA sequence variants can only explain a fraction of the observed variance of complex traits, and this seems also true when single variants are used as moderators in GxE analyses. Of course, this does not invalidate the general GxE concept, but it does raise the question of how the “gene” side of the equation can be modeled given the highly polygenic architecture of complex traits. One way forward is to use polygenic scores, which reflect the genetic predisposition for traits or disorders in a single summary metric by weighting the effect sizes of many variants across the genome. Here, we review the use of polygenic scores in GxE research and highlight their potential to provide important insights into the complex interplay between genetics and the environment in the development of mental health problems.

## 2 The concept of polygenic scores

A precondition for creating polygenic scores is the availability of a genome-wide association study (GWAS) of the trait or disorder of interest. GWAS of mental disorders and behavioral traits have increased vastly in sample size over the past two decades. One factor contributing to this growth is the increasing availability and decreasing cost of genotyping technology, which has made it possible to genotype large numbers of individuals at a relatively low cost. This has allowed researchers to include more participants in studies, thereby increasing statistical power and the likelihood of detecting associations between genetic variants and mental health traits. Larger GWAS sample sizes have also increased the predictive value of polygenic scores derived from these GWAS results.

Over the last decade, GWAS have revealed how inherited variations contribute to numerous complex diseases and disorders. Many mental disorders (e.g. depression, post-traumatic stress disorder) and other common diseases (heart disease, cancer) have a genetic basis that is highly polygenic, composed of thousands of genetic variants each conferring a tiny increment of risk (6). This has led to the recognition that mental disorders and behavioral traits are influenced by the combined effects of many genetic and environmental factors, rather than any single genetic variant or environmental exposure.

**Figure 1:**
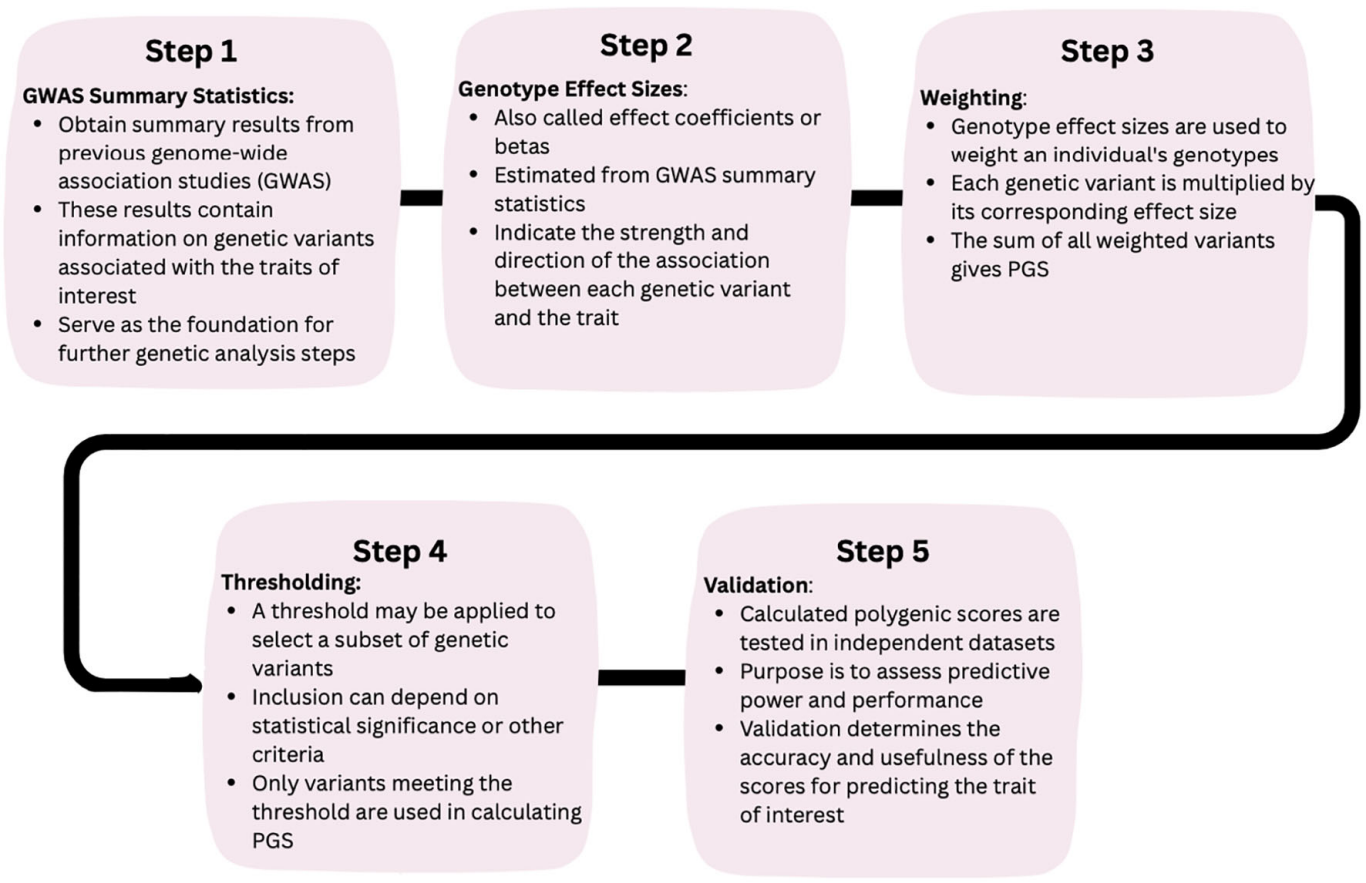
How to calculate PGS?

Despite advances in characterizing the genetic architecture of traits and disorders, GWAS results are not an endpoint but rather a starting point for further research (7). In addition to gene discovery, GWAS findings open several avenues for follow-up investigations. Notably, GWAS summary statistics can be leveraged to create aggregate genetic risk scores that reflect an individual’s genetic disposition for a trait or disease in a single number, which can then be used in downstream studies as predictors or moderators. These scores have been variously called polygenic risk scores (PRS), genome-wide polygenic scores (GPS), or simply polygenic scores (PGS), and we will use the term PGS here. The main steps involved in calculating PGS from GWAS summary statistics are illustrated in **Figure 1**.

Taken together, the use of polygenic scores is the most common method for combining information from many loci. PGS have been used to associate genetic disposition with traits of interest mostly in “main effect” approaches that quantify the amount of variance in a trait explained by genetic background. However, as outlined above, genetic and environmental risks interact in the pathways leading to disorder. Therefore, beyond testing for direct genetic effects, another use of PGS is to test for genetic moderation of environmental risk.

To provide a structured synthesis of the literature, reviewed studies are organized according to the type of environmental exposure under investigation rather than by outcome or genetic approach alone. This exposure-based framework reflects the central premise of gene–environment research, namely that the nature, timing, and measurement of environmental stressors critically shape the detectability and interpretation of interaction effects. Accordingly, the review progresses from acute and time-limited stressors with clearly defined onset, to early-life and social-interpersonal environments, followed by cumulative and traumatic exposures, genome-wide approaches to stress sensitivity, and finally psychological intervention as a controlled environmental condition. This sequence is intended to guide the reader from more precisely operationalized exposures toward broader, more complex, and indirectly modeled environments, thereby highlighting how methodological features of exposure assessment influence patterns of findings across studies (**Table 1**).

**Table 1:** Summary of studies examining gene–environment interactions using polygenic scores (PGS) across mental health and treatment outcomes.

PGS in time-limited stress exposures in adulthood						
#	Author name	Aim/hypothesis	Methodology	PGS used	Phenotype investigated	Findings
1	(Fang, Scott, Song, Burmeister, Sen (8))	Test whether a polygenic risk score for major depressive disorder (MDD-PGS) can predict depression development under predictable stress (physician internship).	5,227 medical interns of European ancestry (mean age 27.6, 50.3% women). Prospective cohort. PHQ-9 depression scores measured at baseline and every 3 months during internship year.	MDD-PGS, based on GWAS meta-analysis (PGC, UK Biobank, 23andMe).	Depressive symptoms measured by PHQ-9; self-reported depression (PHQ-9 ≥10).	MDD-PGS predicted significantly higher depression symptoms under stress (internship) compared to baseline (β = 0.095, p = 4.7×10^−16^ vs β = 0.063, p = 5.4×10^−6^). Low PGS identified resilient individuals. Effect partially mediated by neuroticism, previous depression, early environment.
2	(Peter et al. (9))	Test if PGS for depression and neuroticism predict perceived stress trajectories during a long academic stress period vs. controls.	Prospective-longitudinal quasi-experimental study of 432 law students (196 with cortisol data). Repeated ambulatory stress assessments over ~13 months.	DEP-PGS (depression) and NEU-PGS (neuroticism).	Perceived stress (momentary, daily life), depression symptoms, cortisol awakening response parameters (AUCg, AUCi).	DEP-PGS showed no association with stress trajectories; NEU-PGS predicted baseline perceived stress and steeper increases under academic stress but no effect on depression symptoms or cortisol response.

Early-life family and caregiving environments						
#	Author name	Aim/hypothesis	Methodology	PGS used	Phenotype investigated	Findings
3	(Nelemans et al. (10))	Examine whether polygenic risk for major depression interacts with parental criticism to predict trajectories of adolescent depressive symptoms.	6-year longitudinal community study of 327 Dutch adolescents (baseline age ≈13) using latent growth models and multi-informant parental criticism indices.	PGS for major depression (Hyde et al., (31)), with sensitivity analyses at varying thresholds.	Depressive symptoms across adolescence (intercept and slope).	Higher PGS associated with elevated depressive symptoms, with stronger effects under high parental criticism — supporting a gene × environment interaction.
4	(He & Li (11))	Test whether ADHD-PGS interacts with childhood maltreatment in predicting ADHD symptoms.	4,722 individuals of European ancestry from Add Health Study (longitudinal US cohort). Retrospective self-report of childhood ADHD symptoms and maltreatment.	ADHD Polygenic Score (Demontis et al., (32) GWAS).	Childhood ADHD symptoms (self-reported ages 18–26).	ADHD-PGS and maltreatment were both independently associated with more ADHD symptoms. No significant interaction; low ADHD-PGS didn’t buffer against maltreatment effects. Exposure and vulnerability constructs are closely related.
5	(Trotta, Iyegbe, Forti, et al. (12))	Test whether the effect of childhood adversity on risk of first-presentation psychosis is moderated by the polygenic risk score for schizophrenia.	Pilot study; 80 first-episode psychosis patients and 110 controls; PRS from PGC data; childhood adversity assessed with CECA.Q.	PGS for schizophrenia (PGC data).	First-presentation psychosis (schizophrenia-spectrum and affective psychoses).	Both higher schizophrenia PGS and childhood adversity independently predicted psychosis, but no significant G×E interaction; effects additive. Childhood adversity was assessed retrospectively.

Social and interpersonal stress in adulthood						
#	Author name	Aim/hypothesis	Methodology	PGS used	Phenotype investigated	Findings
6	(Cleary, Fang, Zahodn, Bohnert, Burmeister, Sen (13))	Test whether social support moderates the effect of genetic liability (depression PGS) on depressive symptoms during periods of major life stress.	Two naturalistic cohorts under stress: 1,011 interns (before/during first internship) and 435 recently widowed older adults; longitudinal analyses of PGS × social support interaction.	Polygenic risk score for major depressive disorder (MDD-PGS).	Change in depressive symptoms (survey measures) in response to stress exposure (internship or widowhood).	Significant interaction: individuals with higher PGS were more sensitive to changes in social support (both losses and gains). High PGS individuals benefitted more when social support improved.
7	(Wang, Lifelines Cohort Study, Hartman, Snieder (14))	Investigate whether stress-related exposures amplify the effects of polygenic risk scores (PRSs) for depression and anxiety.	41,810 participants from the Lifelines Cohort Study (Netherlands). Depression and anxiety assessed with MINI interviews. Linear mixed models accounting for familial relatedness.	PGS for depression (Howard et al., 2019) and PGS for anxiety (Purves et al., (33)).	Current depression and anxiety symptoms (MINI sum scores); stress exposures included long-term difficulties, stressful life events, reduced social support, childhood trauma, loneliness.	Nine out of ten PGS × stress exposure interactions were significant. Stress exposures (e.g., loneliness, long-term difficulties) amplified genetic effects on both depression and anxiety. Childhood trauma amplified genetic risk for depression but not anxiety. Strong support for gene–environment interactions.
8	(Colodro-Conde et al. (15))	Test the diathesis–stress model for depression: Does genetic vulnerability (measured via MDD-PGS) interact with stressful life events (SLE) to influence depression risk?	5,221 individuals from 3,083 Australian twin families (European ancestry). Linear mixed models accounting for relatedness; depression assessed via mailed surveys using Item Response Theory (IRT) combined scale. Stressors: personal SLEs, network SLEs, and social support.	Polygenic Risk Score for Major Depressive Disorder (based on updated PGC-MDD GWAS, N = 159,601).	Depression symptom scores (IRT scale); lifetime DSM-IV diagnosis of MDD (subset).	MDD-PGS predicted depression scores (0.46% variance explained, p = 5.01e-08). Significant PGS × personal stressful life events interaction (0.12% variance explained, p = 0.0076), stronger in women. Supports multiplicative diathesis–stress model.

Cumulative life stress and socioeconomic context						
#	Author name	Aim/hypothesis	Methodology	PGS used	Phenotype investigated	Findings
9	(Agerbo et al. (16))	Test whether genetic liability, parental psychiatric history, and socioeconomic status jointly predict risk of early-onset depression.	17,098 patients with depression and 18,582 controls; genetic, clinical, and socioeconomic register data combined.	Polygenic scores for depression.	Early-onset depression diagnosis.	Higher depression PGS, parental psychiatric history, and lower socioeconomic status independently and additively increased risk of early-onset depression. Socioeconomic indicators may reflect both exposure and vulnerability processes, which are difficult to separate analytically.
10	(Musliner et al. (17))	Test whether polygenic liability for depression and stressful life events (SLEs) independently and interactively predict hospital-treated depression in early life.	38,716 individuals (iPSYCH2012 case–cohort study, Denmark). Birth cohorts 1981–2005. Prospective register-based design using Cox regressions.	MDD Meta-PGS (combining internal and external training; based on Howard et al., (32) GWAS).	Early-onset depression diagnosed in psychiatric care (hospital-treated before age 31).	Higher depression PGS (HR = 1.35) and more SLEs (HR = 1.36) independently predicted greater depression risk. Small but significant negative interaction (β = -0.04). Combining PGS and SLE data improved high-risk identification.
11	(Østergaard et al. (18))	Test whether polygenic risk for ADHD and psychosocial environmental factors independently or interactively affect ADHD risk.	33,872 individuals from Denmark (iPSYCH sample): 13,725 ADHD cases and 20,147 controls. Register-based cohort; logistic regression analysis.	Polygenic Risk Score for ADHD (Demontis et al., 2019).	Diagnosis of ADHD (ICD-10 F90.0) from national registers.	Higher ADHD-PGS strongly associated with ADHD risk (OR = 6.03 for top 2%). Psychosocial adversity also linked to higher ADHD risk; no gene–environment interaction.

Trauma and severe adversity						
#	Author name	Aim/hypothesis	Methodology	PGS used	Phenotype investigated	Findings
12	(Peyrot et al. (30))	Test whether childhood trauma (CT) moderates the effect of PGS on the risk of MDD.	Meta-analysis of 9 international cohorts, n = 5,765 (3,024 MDD cases, 2,741 controls). Logistic regression and random-effects meta-analysis.	PGS-MDD (GWAS with ~110,000 individuals); secondary analyses with SCZ and BIP PGS.	Diagnosis of MDD (DSM-IV); childhood trauma (2-domain and 5-domain scales).	MDD-PGS and childhood trauma both independently associated with higher depression risk; no significant interaction found.
13	(Mullins, Lewis, et al. (19))	Examine whether polygenic risk for MDD interacts with environmental adversity to influence MDD risk.	RADIANT and GSK-Munich cohorts; PGS tested for interaction with reported childhood trauma and SLEs.	PGS for MDD (PGC GWAS).	Lifetime diagnosis of MDD.	Both polygenic risk and environmental adversity independently increased MDD risk; no significant gene–environment interaction. Childhood trauma may capture both exposure and vulnerability processes.
14	(Coleman, Peyrot, Purves, et al. (20))	Explore the relationship between genetic risk, major depressive disorder (MDD), and trauma exposure.	126,522 genotyped UK Biobank participants of European ancestry; stratified by reported trauma exposure (n = 24,094–92,957); cross-sectional design.	MDD, SCZ, BIP, BMI, HbA1c (negative control).	Self-reported MDD, trauma exposure, and other phenotypes (e.g., waist circumference, psychiatric disorders, education).	Higher SNP-based heritability of MDD in traumatized individuals (24%) vs. non-traumatized (12%). High genetic correlation between MDD and trauma; significant PGS × trauma interactions on additive scale. Trauma exposure was assessed retrospectively.

GWIS studies						
#	Author name	Aim/hypothesis	Methodology	PGS used	Phenotype investigated	Findings
15	(Arnau-Soler et al. (21))	Identify genetic variants influencing individual differences in stress-sensitivity and their role in MDD.	Genome-wide interaction study using UK Biobank (N = 23,092) and Generation Scotland (N = 7,155), modeling SNP × MDD status on neuroticism.	PGS derived from GWIS of stress-sensitivity (PRS_SS), compared with PGS for MDD and neuroticism.	Neuroticism scores (EPQN) and MDD diagnosis.	No single SNP reached genome-wide significance; ZNF366 achieved genome-wide significance. Stress-sensitivity PGS improved MDD prediction in Generation Scotland sample. Parental disorder and educational indicators may reflect overlapping exposure and vulnerability components.
16	(Suppli, Musliner, et al. (22))	Conduct a genome-wide by environment interaction study (GWEIS) to test whether genetic variants interact with stressful life events (SLEs) to predict hospital-treated depression.	38,716 individuals from the Danish iPSYCH2012 cohort (18,532 depression cases, 20,184 controls). Cox regression modeling; replication in UK Biobank (n = 73,258).	No PGS; GWEIS focused on SNP × SLE interactions.	Hospital-treated MDD (ICD-10 F32–F33); SLEs via national registers (parental death, illness, maltreatment).	Three genome-wide significant SNP × environment interactions identified (ABCC1, AKAP6, MFSD1) but none replicated in UKB. No robust replicable findings.

Polygenic scores as predictors of treatment response						
#	Author name	Aim/hypothesis	Methodology	PGS used	Phenotype investigated	Findings
19	(Wannemüller et al. (23))	Test whether polygenic risk scores predict CBT treatment outcomes in phobias	342 phobic patients; exposure-based CBT (5-session dental fear vs long multi-session mixed phobias); PRS vs remission & fear reduction	EA, IQ, Neuroticism, GAD, MDD, SCZ, ASD, BMI (control)	Treatment outcome (remission, fear reduction, dropout)	EA-PGS strongly predicted better outcomes in short dental-fear CBT (remission, fear reduction, adherence); no PGS effects in long multi-session CBT
20	(Rayner, Coleman, Purves, et al. (24))	Investigate genetic predictors (GWAS, PGS) of outcomes following CBT for anxiety and depression; test for overlap with psychopathology, personality, and learning traits.	Meta-analysis of 3 cohorts (n=2,724): adults with anxiety or MDD and children with anxiety. GWAS and polygenic scoring analyses.	PGS for ADHD, anxiety disorders, ASD, MDD, SCZ, neuroticism, subjective well-being, educational attainment, intelligence.	Therapy outcome measured as change in symptom severity after CBT.	No genome-wide significant SNP associations. Polygenic scores did not significantly predict CBT outcome; nominal links not significant after correction.

## 3 PGS in time-limited stress exposures in adulthood

Time-limited stress exposures in adulthood provide a valuable framework for studying gene–environment interplay, as they allow direct comparison between pre-stress baseline functioning and periods of predictable, externally imposed stress. Two prospective studies have used such paradigms to examine whether polygenic risk scores moderate emotional and stress-related responses under sustained but time-bounded adversity.

Fang et al. (8) measured depressive symptoms using the PHQ-9 in 5,227 medical interns (mean age ~27.6 years, 50.3% women) before the start of their internship year and then every 3 months during the stressful internship year. PHQ-9 scores averaged 2.5 at baseline and 5.6 during the internship; 3.4% met PHQ-9 criteria for depression at baseline, compared to 33.2% at least once during internship. A higher MDD PGS significantly predicted greater risk of developing depression during the internship year. Importantly, a significant GxE was observed: the association between MDD-PGS and depression became stronger under the high-stress condition of the internship (PGS × stress interaction: β = 0.036, p = 0.005). Known non-genetic risk factors contributed significantly less to depression during stress than at baseline, suggesting that the MDD-PGS added its own predictive power under stressful conditions. Notably, interns with a low MDD-PGS were highly resilient, showing substantially lower depression rates despite the stress. These results suggest that the MDD-PGS may help predict vulnerability and resilience under stress.

Peter et al. (9) conducted a prospective, quasi-experimental study of 432 law students over 13 months. Students preparing for a major exam constituted a long-lasting stress group, while a control group of students was not exposed to that exam stress. Depression symptoms, two cortisol awakening response parameters, and perceived stress levels were repeatedly measured via ambulatory assessments. Results showed no relationship between the depression PGS and stress-related measures. However, a robust GxE effect emerged for the neuroticism PGS: higher polygenic risk for neuroticism predicted greater increases in perceived stress up to the exam date, but only among students under exam stress (p < 0.001). In other words, higher neuroticism PGS was associated with steeper stress-level increases in the stressed group, whereas in the no-stress group this effect was absent. At baseline (before the stress phase), higher perceived stress was already linked with higher neuroticism PGS in both groups.

Studies examining time-limited stress exposures indicate that genetic liability is more strongly expressed under conditions of predictable and sustained stress than during non-stress periods. Across these designs, GxE interactions are most evident when stress exposure is externally imposed and temporally well defined, allowing for clear comparisons between baseline and stress phases. The findings further suggest that interaction effects depend on the alignment between the polygenic score and the outcome assessed: polygenic risk for depression predicted depressive symptoms during internship stress, whereas polygenic risk for neuroticism was more closely related to perceived stress trajectories during examination stress. These results point to a degree of specificity in how genetic liability moderates responses to stress, and suggest that time-limited stress paradigms provide a comparatively sensitive context for detecting polygenic moderation of stress-related emotional responses.

## 4 Early-life family and caregiving environments

Early-life family and caregiving environments represent a central context in which genetic liability may shape sensitivity to stress, given their salience for emotional development and the prolonged nature of exposure. From a GxE perspective, these settings allow tests of whether polygenic risk amplifies or attenuates responses to relational adversity during sensitive developmental periods. We identified three longitudinal studies examining interactions between polygenic risk and caregiving-related stressors, ranging from parental criticism to childhood maltreatment, across adolescence and into adulthood.

Nelemans et al. (10) conducted a longitudinal study of Dutch adolescents (baseline age ~13 years, N = 327) over six years. The study tested whether a polygenic risk score for adult major depression (MD) predicted the developmental trajectory of depressive symptoms from early to late adolescence, and whether genetic risk moderated the effect of a specific environmental risk factor (parental criticism) on adolescent depression. Annual assessments of adolescents’ depressive symptoms and mothers’ critical parenting were collected via questionnaires. Findings showed that higher MD genetic risk was associated with elevated depressive symptoms across adolescence, and that lower parental criticism was associated with lower depressive symptoms regardless of polygenic risk level. Importantly, a significant interaction between the MD-PGS and parental criticism (p < 0.05) indicated that adolescents with higher polygenic risk were more sensitive to the effects of high parental criticism, developing greater depressive symptoms when exposed to more critical parenting. In other words, genetic vulnerability to depression predicted higher symptom levels especially among adolescents facing high criticism. These findings provide clear evidence that polygenic risk for depression can predict depressive symptom trajectories in youth, particularly for those exposed to adverse family environments (critical parenting).

He & Li (11) looked at the interaction between childhood/adolescent maltreatment and polygenic risk for ADHD. Prior work suggested that individuals with low ADHD genetic risk tend to have better functional outcomes than those with medium or high genetic risk (in cognitive, mental health, and social domains). The study hypothesized that a low ADHD-PGS might be a protective factor in the context of abuse. Using data from 4,722 participants in the National Longitudinal Study of Adolescent to Adult Health (with both genetic and phenotypic data), the researchers created an ADHD PGS based on the latest GWAS and stratified participants into low, medium, and high PGS groups. A maltreatment factor score was derived from five types of self-reported childhood abuse/neglect experiences. Consistent with expectations, both the ADHD PGS and maltreatment were positively associated with ADHD symptoms. However, there was no evidence of an interaction between ADHD polygenic risk and maltreatment on ADHD symptom outcomes – in other words, high genetic risk did not magnify the effect of abuse on symptom levels, nor did low genetic risk buffer it. This suggests that, in this cohort, genetic liability and maltreatment contributed additively to ADHD risk rather than multiplicatively.

Trotta et al. (12) asked whether an MDD PGS interacts with childhood trauma to predict adult depression. Using data from three large cohorts (ESTRA, UK Biobank, Generation Scotland; combined N > 3,000), the study computed PGS for depression and examined their interaction with self-reported childhood trauma. Linear regression showed that both the PGS and childhood trauma independently predicted higher depressive symptoms (p < 0.001 for each main effect), but there was no significant PGS × trauma interaction on depression (p > 0.05). In other words, individuals with higher genetic risk and those with trauma history were more depressed on average, but genetic risk did not significantly modify the effect of trauma. These findings suggest that while genetic predisposition and early-life adversity each contribute to depression risk, the PGS did not noticeably moderate the impact of trauma in those cohorts.

Across the three studies reviewed, evidence for gene–environment interaction in early-life family contexts was mixed. A significant interaction emerged only in the study focusing on a specific, relational caregiving stressor (parental criticism), where higher polygenic risk was associated with greater sensitivity to adverse parenting. In contrast, studies examining broader or retrospectively assessed forms of childhood maltreatment reported additive effects of genetic risk and adversity, but no interaction. These findings suggest that, at least in the limited literature available, genetic moderation is more readily detected in longitudinal designs targeting proximal, relational caregiving exposures than in studies relying on aggregated or temporally distant adversity measures.

## 5 Social and interpersonal stress in adulthood

This section reviews three studies examining how social and interpersonal stressors in adulthood dynamically modify the expression of polygenic risk for depression and anxiety, particularly through variations in social support and interpersonal strain.

Cleary et al. (13) investigated how genetic risk for major depression (MDD-PGS) interacts with social support to predict depressive symptom trajectories during significant life stress. The study used two longitudinal cohorts: 1,011 medical interns from the *Intern Health Study* and 435 recently widowed older adults from the *Health and Retirement Study*. Depressive symptoms and social support were assessed before and after stress exposure using validated scales (PHQ-9 and CES-D for depression; MSPSS and LBQ for social support). Results showed a significant interaction between MDD-PGS and changes in social support in both cohorts. These findings indicate that individuals with higher genetic risk are more sensitive to social support fluctuations, showing increased depression when support decreases but greater resilience when it strengthens (consistent with a differential susceptibility model of gene–environment interaction).

Wang et al. (14) investigated whether exposure to different types of stressors relate to depression and anxiety, and whether these exposures reinforced genetic susceptibility to symptoms of depression and anxiety. A sample of 41,810 individuals with available genome-wide genotypes was assessed for current depression and anxiety symptoms using the MINI Neuropsychiatric Interview. Long-term difficulties, stressful life events, low social support, childhood trauma, and loneliness were quantified as stress exposures via self-report questionnaires. Results showed that PGS-by-environment interaction effects were significant in 9 out of 10 comparisons between the two PGS (for depression and anxiety) and the five stress-related exposures. Specifically, interactions involving lack of social support, increased long-term difficulties, stressful life events, loneliness, and reduced social support indicated that genetic risk amplified the effects of these stressors on depression and anxiety outcomes. Childhood trauma exposure was significantly interactive with the PGS for depression (P = 1.78 × 10^−5^), but not with the PGS for anxiety (P = 0.32).

Colodro-Conde et al. (15) tested a diathesis-stress model for MDD by examining whether an MDD polygenic score interacted with life stressors. Data were drawn from an Australian twin registry, where stress was measured via self-reported personal and network life events. A linear mixed model showed that both the MDD PGS (p = 5.0 × 10^−8^; variance explained ≈ 0.5%) and personal stressors (p < 0.001; variance explained ≈ 12.9%) were significant predictors of depression symptomatology. A modest but significant interaction between the PGS and personal stressors (p = 0.0076; variance explained ≈ 0.1%) supported the diathesis-stress model: individuals with high genetic susceptibility had higher depression scores under high stress. No significant interaction was found between PGS and network stressors or social support in that study. While these results support the notion of GxE in the development of depressive symptoms, the very small variance explained by PGS × stress interaction argues against using genetic risk alone for clinical prediction. Nevertheless, the study provided empirical evidence for the diathesis-stress model of depression by demonstrating a significant PGS–stress interaction in predicting depressive symptoms.

In contrast to studies emphasizing discrete or cumulative stress exposure, research on social and interpersonal stress in adulthood consistently highlights the role of social context as a dynamic modifier of genetic liability. Across these studies, a recurrent pattern emerges whereby polygenic risk for depression or anxiety is more strongly expressed under conditions of reduced social support, interpersonal strain, or social isolation. Interaction effects are most likely to be replicated when social exposures are assessed with sufficient granularity and temporal sensitivity, particularly when changes in social support are captured before and after stress exposure. Compared to broader adversity measures, social and interpersonal stressors appear to produce more consistent GxE interactions, possibly because they directly shape emotional regulation processes in daily life.

## 6 Cumulative life stress and socioeconomic context

This section reviews three large-scale register-based studies examining whether cumulative life stress and socioeconomic adversity interact with polygenic liability for depression or ADHD, or instead contribute largely additively to psychiatric risk.

Agerbo et al. (16) examined a subcohort of the Danish iPSYCH sample, a case-cohort including all singletons born in Denmark between 1981 and 2005. The analysis included individuals diagnosed with depression (n = 17,098) and controls (n = 18,582). The study quantified the association between a depression PGS and the absolute risk of early-onset MDD, and also evaluated non-genetic risk factors (sex, parental socioeconomic status, parental history of mental disorders), as well as their joint effects with PGS. The absolute risk of depression by age 30 was 8.1% among individuals in the highest 2% of the PGS distribution, compared to 2.7% among those in the lowest 2%. There was no evidence of statistical interaction between the PGS and any single non-genetic risk factor. However, risk subgroups could be identified by combining genetic and psychosocial risk factors. For example, women in the top 2% of the PGS distribution whose both parents had a history of mental illness had a 14.6% absolute risk of a depression diagnosis by age 30, compared with 4.4% in women with low PGS and no parental illness. This highlights how PGS may be used alongside family history and other factors to stratify risk.

In the Danish iPSYCH2012 study, Musliner et al. (17) used a case–cohort design of over 38,000 participants and examined how a depression PGS and prospectively assessed stressful life events (SLEs) jointly predict risk for early-onset, clinically treated depression. Both genetic liability and cumulative SLE exposure were robust independent predictors, each conferring approximately 35% increased risk per standard deviation or additional event. The interaction between PGS and SLEs was statistically detectable but small, negative on the multiplicative and slightly positive on the additive scale, indicating that the combined effects were not substantially greater than expected from the sum of their independent contributions Consequently, the authors conclude that there is minimal evidence for a clinically meaningful GxE interaction in this context. Despite modest statistical interaction, absolute risk stratification for depression incidence ranged from ~1.5% in low-risk males (lowest PGS, no SLEs) to ~19% in high-risk females (highest PGS, ≥ 4 SLEs). These findings show that PGS and environmental adversity each contribute meaningfully and largely additively to depression risk, supporting a diathesis–stress framework with limited evidence for synergistic G×E effects but considerable potential for risk stratification and early prevention.

Østergaard et al. (18) investigated whether the psychosocial environment and polygenic liability for ADHD influence ADHD risk independently or interactively. In a Danish register-based cohort of 13,725 individuals with ADHD and 20,147 controls, the ADHD PGS was a strong predictor of ADHD status (odds ratio ~6 for top 2% vs bottom 2% of PGS). Several psychosocial risk factors at birth (parental history of mental disorders, low parental education, unemployment, low income) were each associated with increased risk of ADHD. Crucially, no statistically significant interactions were found between the ADHD PGS and any of the measured psychosocial factors on ADHD risk (all p > 0.05). In other words, genetic liability and these environmental factors each influenced ADHD risk but did not appear to moderate each other. The absence of GxE in this large sample reinforces the idea that, for ADHD, current PGS capture mainly main effects and any interaction with typical psychosocial variables may be too small to detect.

Across these three studies, evidence for GxE in the context of cumulative life stress and socioeconomic adversity was limited. Polygenic liability and psychosocial risk factors consistently showed independent associations with depression or ADHD risk, whereas interactions between genetic risk and cumulative or distal environmental exposures were small or absent. Where statistical interactions were detectable, their effect sizes were modest and of uncertain clinical relevance. Nonetheless, combining genetic liability with cumulative environmental risk allowed meaningful stratification of absolute disorder risk. Together, these findings suggest that, in large register-based cohorts, cumulative stress and socioeconomic adversity primarily contribute additively to mental health outcomes, with GxE interactions appearing less prominent than in studies of more proximal or precisely defined stressors.

## 7 Trauma and severe adversity

This section reviews three large and influential studies examining whether exposure to trauma or severe adversity moderates the association between polygenic liability and risk for depression.

Peyrot et al. (30) conducted a meta-analysis of nine Psychiatric Genomics Consortium (PGC) cohorts (3024 MDD cases and 2741 controls) to test whether childhood trauma (CT) moderates the effect of a MDD-PGS on disease risk, using both a 2-domain (sexual or physical abuse) and a 5-domain CT measure (sexual abuse, physical abuse, emotional abuse, emotional neglect, physical neglect). The MDD-PGS, derived from a discovery GWAS of ≈110 000 individuals, was significantly associated with MDD (OR = 1.24, p = 3.6 × 10^−5^) and CT was strongly linked to MDD (OR = 2.63, p = 3.5 × 10^−18^ for the 2-domain measure; OR = 2.62, p = 1.4 × 10^−5^ for the 5-domain measure) . However, interaction analyses revealed no evidence that CT moderates the PGS effect: the PGS × 1-domain interaction yielded OR = 1.00 (p = 0.89) and the PGS × 5-domain interaction yielded OR = 1.05 (p = 0.66) . Consequently, the previously reported significant but opposite interaction effects in the NESDA and RADIANT UK cohorts appear to have been chance findings, and the heterogeneity of MDD is unlikely to be driven by genome-wide genetic moderation by childhood trauma . Overall, the study provides the largest to-date test of PGS-CT interaction in MDD and concludes that, despite robust main effects of both genetics and trauma, their combined effect does not deviate from additivity.

Results from Mullins et al. (19) were consistent with the above. This study used depression cases (n = 1,605) from prior research to examine interactions between stressful life events or childhood trauma and polygenic risk in MDD etiology. As expected, childhood trauma and stressful life events were each strongly associated with MDD (p = 2.19 × 10–^4^ and p = 5.12 × 10^-20^, respectively). However, no interaction was found between the PGS and stressful life events on depression risk. There was an interaction between PGS and childhood trauma (p = 0.002), but it was in an unexpected direction: individuals with the most severe childhood trauma had lower depression polygenic scores on average. This paradoxical finding suggests possible sampling effects or complexities in how extreme trauma relates to genetic risk (e.g. individuals with extreme trauma might develop depression even with lower genetic loading). In sum, Mullins et al. (19) and other null findings highlight that many early GxE studies using PGS did not detect clear interaction effects, pointing to the need for larger samples and improved measures.

Coleman et al. (20) explored gene–environment interplay by examining depression risk in a UK Biobank subsample of 73,258 participants with or without self-reported trauma exposure. The SNP-based heritability of MDD was substantially higher (h² ≈ 24%) among individuals who reported trauma exposure than among those who did not (h² ≈ 12%). Simulations indicated this difference was not due to confounding by genetic correlation between MDD and trauma exposure. Instead of using a PGS as the moderator, this study directly tested GxE at the genome-wide level by stratifying the heritability estimate. The results imply that genetic contributions to MDD are amplified in the presence of trauma exposure. While the difference in SNP-heritability between the trauma-exposed and non-exposed groups was highly significant (p < 0.001), the genetic correlation between MDD cases with trauma and those without trauma was not significantly different from 1.0. This suggests that the same genetic risk variants for MDD are at play, but their aggregate effect is stronger when traumatic experiences are present.

Across the three studies reviewed, evidence for polygenic moderation in the context of trauma and severe adversity was limited. Genetic liability and trauma exposure showed robust and largely independent associations with depression risk, while systematic PGS × trauma interactions were generally absent. Findings from meta-analytic and large cohort analyses suggest that combined effects largely follow additive models, with little indication of multiplicative interaction. These results imply that severe or heterogeneous trauma exposures may reduce sensitivity to detect differential genetic effects, despite strong main effects of both genetic liability and environmental adversity.

Taken together, among the reviewed studies operating within a diathesis-stress framework, most studies operationalized exposures as stressful life events, trauma, or socioeconomic adversity, and vulnerabilities as enduring individual characteristics such as polygenic liability or prior psychopathology. In contrast, resources were rarely included as explicit model components, with only a small number of studies modelling social support directly. Developmental timing was also inconsistently addressed, with relatively few studies embedding gene by environment interplay within a lifespan framework (**Table 2**).

**Table 2:** Operationalization of diathesis–stress model components across reviewed studies.

Study (author, year)	Exposure (E)	Vulnerability (V)	Distress/outcome (D)	Resources (R)	Developmental timing
(Fang et al. (8))	Medical internship (time-limited occupational stress)	MDD polygenic score	Depressive symptoms (PHQ-9)	Not modelled	Young adulthood
(Peter et al. (9))	Examination stress in law students	Neuroticism PGS	Perceived stress, cortisol awakening response	Not modelled	Young adulthood
(Nelemans et al. (10))	Parental criticism	MDD polygenic score	Depressive symptoms	Implicit (low criticism)	Adolescence
(He & Li (11))	Childhood/adolescent maltreatment	ADHD polygenic score	ADHD symptoms	Not modelled	Childhood/adulthood
(Trotta et al. (12))	Childhood trauma	MDD polygenic score	Depressive symptoms	Not modelled	Early adversity to adulthood
(Cleary et al. (13))	Stress exposure + change in social support	MDD polygenic score	Depressive symptoms	Social support (explicitly modelled)	Adulthood
(Wang et al. (14))	Life events, loneliness, low social support	Depression/anxiety PGS	Depression and anxiety symptoms	Social support (explicit)	Adulthood
(Colodro-Conde et al. (15))	Personal life stressors	MDD polygenic score	Depressive symptoms	Not modelled	Adulthood
(Agerbo et al. (16))	Socioeconomic adversity, parental mental illness	Depression PGS	Clinical depression diagnosis	Socioeconomic indicators (risk-focused)	Birth/adulthood
(Musliner et al. (17))	Cumulative stressful life events	Depression PGS	Hospital-treated depression	Not modelled	Adolescence/adulthood
(Østergaard et al. (18))	Psychosocial adversity at birth	ADHD polygenic score	ADHD diagnosis	Not modelled	Early life
(Peyrot et al. (30))	Childhood trauma (multi-domain)	MDD polygenic score	MDD diagnosis	Not modelled	Early adversity
(Coleman et al. (20))	Trauma exposure	Genome-wide liability	Depression (heritability stratification)	Not modelled	Lifespan
(Mullins et al. (19))	Childhood trauma; stressful life events	MDD polygenic score	Major depressive disorder	Not modelled	Childhood adversity/adulthood

## 8 GWIS studies

The majority of studies investigating gene–environment interplay have used PGS for mental disorders as predictors in GxE models. It would be desirable to have a genetic measure of *environmental sensitivity* itself; however, the lack of comprehensive, reliable environmental data in large-scale genetic studies makes this challenging.

As a first step in that direction, Arnau-Soler et al. (21) followed a genome-wide interaction study (GWIS) approach to derive a proxy measure of “stress sensitivity.” They searched for genetic variants whose association with neuroticism differed depending on depression status (higher neuroticism only in individuals with MDD, but not in controls). Analysis of the UK Biobank (N = 23,092) and Generation Scotland (N = 7,155) data did not reveal any SNP interactions reaching genome-wide significance. However, a gene-based analysis identified one genome-wide significant gene, *ZNF366*, which is involved in regulation of the glucocorticoid receptor, a key regulator of the hormonal stress response system. In a second step, the authors constructed a polygenic score for “stress sensitivity” based on the top GWIS findings and tested it in independent samples. In models that included polygenic risk scores for both MDD and neuroticism (from standard GWAS), adding the stress-sensitivity PGS from the GWIS significantly improved the prediction of depression in the Generation Scotland cohort.

In a similar approach, the same group (25) performed a GWIS of depressive symptoms and stressful life events in the two UK cohorts. Again, only a limited number of suggestive GxE loci were identified. However, a polygenic score derived from this depressive-symptoms GWIS significantly improved the prediction of depressive symptom scores in an independent sample (25), echoing the idea that incorporating interaction effects can enhance polygenic predictions modestly.

Suppli et al. (22) conducted a GWIS study focusing on stressful life events (SLEs) in relation to hospital-treated depression. Using data from 18,532 depression cases and 20,184 controls in the iPSYCH2012 Danish sample, the researchers examined how genetic predispositions combined with stressful experiences affect depression risk. SLE exposures (e.g. family disruption, serious illness, parental disability, child maltreatment) were ascertained from national registry records. The GWIS analysis in the Danish sample yielded three genome-wide significant GxE loci for depression risk: *ABCC1* (rs56076205, p = 3.7 × 10^-10^), *AKAP6* (rs3784187, p = 1.2 × 10^-8^), and *MFSD1* (rs340315, p = 4.5 × 10^-8^). These loci could represent genetic factors influencing vulnerability to stress-related depression. However, when the top hits were tested in a replication sample (22,880 depression cases, 50,378 controls from UK Biobank), none of the SNPs replicated (all p > 0.7). The lack of replication raises questions about the validity of the initial findings. In the end, this large study concluded that no definitive gene–stress interactions could be confirmed in their population. The authors underscored the need for even larger GWIS samples and more precise phenotyping to achieve adequate statistical power, noting that their results contribute to ongoing debates about the complex relationship between genetic susceptibility and environmental impacts on depression.

Across the studies reviewed, efforts to move beyond standard PGS × environment models toward genome-wide interaction approaches yielded mixed results. While GWIS analyses identified few robust or replicable single-variant interaction effects, polygenic scores derived from interaction-based models provided modest but incremental improvements in the prediction of depressive outcomes beyond conventional PGS. Findings were generally constrained by limited statistical power, imprecise environmental measurement, and poor replication of individual loci. Taken together, this literature suggests that interaction-informed polygenic approaches may offer small gains in risk prediction, but that reliably capturing genetic sensitivity to environmental stress will require substantially larger samples and more precise assessment of environmental exposures.

## 9 Psychological intervention as a controlled “environment” - polygenic scores as predictors of treatment response?

In gene–environment research, the environment can be conceptualized in multiple ways, ranging from early-life adversity to cumulative or chronic stress exposures in adulthood. While ethical constraints preclude the experimental induction of harmful environments, it is feasible to systematically introduce beneficial environments. Psychological interventions, such as psychotherapy, therefore offer an ethically tractable and conceptually appealing model of a controlled environmental exposure (26). Within this framework, genetic differences may shape individuals’ responsiveness to treatment, analogous to differential sensitivity to adverse contexts.

From a clinical perspective, identifying predictors of treatment response is of substantial interest, as only a subset of patients benefit from first-line psychological interventions. Even modest improvements in response prediction could reduce time to recovery, limit exposure to ineffective treatments, and improve overall outcomes. Polygenic scores may offer one potential tool for stratifying treatment response or non-response, although their clinical utility remains uncertain (27). Using genetic information in this way might shorten the trial-and-error period to find an effective intervention, enhance overall treatment response rates, and reduce exposure to ineffective treatments (and their side effects), yielding benefits for patients and healthcare systems.

Rayner et al. (24) examined the heritability of response to cognitive behavioral therapy (CBT) using GWAS meta-analyses of symptom change across three samples: adults with anxiety disorders (n = 972), adults with major depressive disorder (n = 832), and children with anxiety disorders (n = 920). Despite being one of the largest genetic studies of psychotherapy response to date (total N = 2,724), the analyses identified no genome-wide significant SNPs, no significant SNP-based heritability of treatment response, and no meaningful genetic correlations between therapy outcome and related traits. These findings suggest that common genetic variants detectable with current GWAS approaches do not exert strong, generalizable effects on CBT response.

An alternative strategy has been to test whether established polygenic scores for psychiatric disorders or related traits predict treatment outcomes. Wannemüller et al. (23) applied this approach in a sample of 342 patients undergoing exposure-based CBT for phobias. In patients with dental phobia receiving a brief, highly standardized treatment, a higher polygenic score for educational attainment was associated with several short-term outcomes, including remission, symptom reduction, and treatment adherence. No comparable effects were observed in a more heterogeneous phobia group receiving longer treatment. These findings suggest that polygenic effects may be detectable under specific conditions, particularly in brief, circumscribed interventions, but require replication and careful interpretation, especially given the lack of long-term predictive effects.

Taken together, the role of polygenic variation in moderating response to psychological intervention has so far been investigated only to a limited extent. To date, evidence consists of a single genome-wide study that did not identify robust genetic influences on psychotherapy response, alongside one additional study suggesting context-specific associations between established polygenic scores and short-term treatment outcomes. Together, these findings indicate that any polygenic moderation of treatment response is likely to be modest and highly dependent on intervention characteristics, outcome definitions, and timing of assessment.

## 10 Discussion

Gene–environment interactions play a crucial role in understanding the etiology of mental health problems. With the advent of large-scale GWAS, it has become possible to aggregate many small genetic effects into single scores capturing polygenic liability for various traits and disorders. These polygenic scores can be used not only for direct prediction of phenotypes but also to investigate how genetic predisposition moderates the impact of environmental risk factors. In this review, we focused on studies examining interactions between PGS and psychological stress exposures, primarily with depression outcomes but also including other conditions like anxiety and ADHD.

Across the studies reviewed, a wide range of environmental exposures has been investigated, from childhood trauma and adversity to more proximal stressors such as work-related stress, recent life events, and social factors like parenting behavior and social support. The overall pattern reveals considerable heterogeneity in findings. Several studies provide evidence supporting the classic diathesis–stress model, showing that genetic liability (captured by PGS) interacts with environmental stressors to predict depression and related outcomes. Particularly robust examples come from studies with well-defined stress exposures and prospective designs, such as medical internship stress or examination stress in students. These studies benefit from temporally precise exposure windows, repeated measurement, and reduced recall bias, which together may help reveal GxE effects that are obscured in other designs. These findings align with long-standing insights from diathesis–stress theory that interaction effects are most likely to be observed when stressors are conceptually precise and developmentally proximal, and may be diluted when vulnerability and stress are broadly defined or aggregated (2).

However, many studies indicate that genetic predisposition and environmental factors contribute independently to psychological outcomes, without significant interaction effects. This inconsistency likely reflects methodological variation across studies, including differences in sample size, statistical power, ancestry composition, and the precision of environmental assessment. Notably, many studies operationalized exposure using summed self-reported life event measures, which, while often reliable, raise concerns regarding validity, including recall bias, mood-congruent reporting, and the aggregation of heterogeneous events that differ in prevalence and outcome relevance. In addition, several studies conflated conceptual components of the diathesis–stress model by treating variables such as childhood trauma, prior psychopathology, socioeconomic status, or parental mental illness as exposures, despite their potential role as enduring vulnerabilities or resources, particularly in adult samples. Registry-based indicators, although prospectively recorded, often capture only extreme or easily codified events and may miss subjective stress experiences. Additionally, differences in PGS construction (e.g., discovery GWAS, thresholds, ancestry alignment) complicate cross-study comparisons and may contribute to variability in detected interaction effects.

A further challenge lies in the complexity of gene–environment interplay itself, which may involve multiple mechanisms beyond simple moderation. For example, gene–environment correlation (rGE) can confound GxE findings: individuals with certain genetic predispositions may be more likely to select, evoke, or perceive stressful environments, making it difficult to distinguish genuine moderation from exposure selection effects. Similarly, the scale on which interactions are modeled matters. Many studies test interactions on the multiplicative scale, which often yields null results, while additive-scale interactions, arguably more relevant for public health, sometimes reveal modest combined effects. Yet even when statistically significant, these interactions typically account for less than 0.2% of the variance, highlighting the gap between statistical significance enabled by large sample sizes and clinical significance, which requires effect sizes that meaningfully change prediction or intervention strategies.

A further conceptual issue concerns the interpretation of polygenic scores within classical diathesis–stress frameworks. In traditional formulations, a diathesis refers to a latent vulnerability that is mechanistically linked to disorder-specific pathways and is activated under particular environmental conditions (2). By contrast, current polygenic scores index aggregated genetic liability derived from genome-wide association studies and capture diffuse, probabilistic risk across thousands of variants with small effects. As such, PGS should not be equated with a diathesis in the classical sense.

Importantly, diathesis–stress models assume some degree of specificity between vulnerability and stressor, whereas most PGS reflect broad liability that overlaps across phenotypes and mechanisms. This lack of specificity may partly explain why many PGS × environment studies yield additive rather than multiplicative effects: a broadly defined genetic score may increase overall risk across environments without conferring heightened sensitivity to particular stressors. From this perspective, null interaction findings do not necessarily contradict diathesis–stress theory, but rather highlight a conceptual mismatch between classical notions of diathesis and the current operationalization of genetic liability via PGS.

Related frameworks such as differential susceptibility and vantage sensitivity further underscore this point by proposing that genetic factors may index general environmental responsiveness rather than vulnerability per se (28, 29). Whether polygenic scores capture such plasticity remains an open empirical question and likely depends on the alignment between the genetic construct, the environmental exposure, and the outcome assessed. Together, these considerations suggest that PGS-based G×E findings should be interpreted cautiously and situated within a broader conceptual landscape that distinguishes between vulnerability, sensitivity, and general liability.

The genome-wide interaction study (GWIS) approach represents an innovative attempt to identify genetic variants specifically involved in stress sensitivity, potentially offering more targeted tools for GxE research. While these efforts (e.g., 21, 25) identified a few promising candidate variants and demonstrated that a stress-sensitivity PGS can modestly improve risk prediction, replication challenges remain substantial. The lack of consistent replication for GWIS hits underscores the need for larger samples, more accurate and granular environmental measures, and methodologies that explicitly model rGE and environmental structure.

In the context of treatment response, emerging research on PGS as predictors of therapeutic outcomes offers a novel application of genetic data. Conceptualizing psychotherapy as a standardized, beneficial environmental intervention allows for controlled tests of environmental sensitivity. Although findings remain preliminary, certain PGS, for example, for educational attainment or related cognitive traits, have been linked to treatment outcomes in specific phobia, whereas large GWAS of CBT response show no robust single-SNP or PGS effects. These mixed results suggest that while PGS may eventually contribute to personalized treatment approaches, they currently lack the predictive power and robustness required for clinical application.

Research on polygenic scores has undoubtedly contributed valuable insights into the genetic architecture of psychological disorders. Some studies demonstrate the potential of PGS in identifying individuals at elevated risk for early-onset disorders (such as those most vulnerable to developing depression), and others hint at using PGS to flag individuals less likely to respond to first-line treatments. However, the clinical utility of these scores remains limited due to modest effect sizes, substantial uncertainty at the individual level, and the context-dependent nature of GxE effects. Unlike monogenic conditions where genetics can directly inform treatment, psychiatric disorders are influenced by complex interactions among genetic, developmental, and environmental factors, making prediction inherently probabilistic rather than deterministic.

Several additional limitations hinder the widespread clinical use of PGS. First, most scores are derived from GWAS conducted in predominantly European-ancestry samples, which limits their generalizability and risks exacerbating health disparities when applied to diverse populations. Second, existing PGS models are static snapshots of genetic liability and do not capture the dynamic interplay between genes and the shifting environments people experience over time. In some cases, environmental exposures, especially severe or chronic adversity, can overshadow polygenic influences, suggesting that models integrating environmental histories with genetic risk will be crucial for capturing true vulnerability.

Although psychological distress is a multidimensional construct, the present review was constrained by the outcome definitions used in the existing polygenic score G×E literature. The reviewed studies predominantly focused on internalizing phenotypes, especially depressive and anxiety symptoms. Anger, despite its relevance as an indicator of distress and stress responsivity with important implications for health and behavioral outcomes, has been largely neglected in this field. Future research would benefit from applying a more comprehensive and theoretically grounded framework of psychological distress more systematically, encompassing anger, externalizing symptoms, and broader stress-related and behavioral outcomes.

## 11 Conclusion and future directions

Taken conclude, polygenic scores have made it possible to test gene–environment interplay using a realistic model of psychiatric genetics, but the empirical signal is context-dependent and generally small. Across the literature reviewed, PGS and stress exposures show robust main effects, while PGS *by* stress interactions are most consistently detected in designs with well-defined, proximal, and temporally bounded exposures (e.g., internships/exams, changes in social support), and are less consistently observed for broad, cumulative, or heterogeneous adversities (e.g., socioeconomic context, retrospective maltreatment/trauma), where effects tend to be largely additive.

To improve the utility of PGS in the mental health field, future research should prioritize the integration of genetic data with detailed, temporally precise environmental assessments. Longitudinal designs that combine genomics with ecological momentary assessment, digital phenotyping, or wearable sensor data could illuminate how genetic risk unfolds in real-life contexts and how individuals dynamically respond to stress. Expanding GWAS to include more ancestrally diverse populations will be essential for improving accuracy, equity, and translational relevance.

Methodological innovation will also be critical. New approaches that incorporate GxE directly into score construction (e.g., GxE-informed PGS, multi-trait GxE models), alongside machine learning techniques that can detect non-linear or higher-order interactions, may improve predictive performance. Coupling polygenic data with intermediate biological measures, such as epigenetic modifications, neuroendocrine stress markers, or neuroimaging phenotypes, may help reveal mechanistic pathways through which genetic susceptibility translates into stress-related psychopathology.

While PGS have significantly advanced GxE research in psychiatry, their clinical role remains uncertain. Current evidence suggests that genetic predisposition explains part of the variance in mental health outcomes, but environmental factors contribute equally or more. Future work should aim to refine polygenic models by integrating multidimensional data and capturing more nuanced GxE dynamics. Until such advances are realized, PGS should be viewed primarily as powerful research tools rather than direct clinical predictors. Nonetheless, integrating polygenic scores with environmental context holds promise for developing more holistic, mechanistic models of mental illness risk and resilience, an endeavor that will require larger, more diverse datasets and a sustained focus on the interplay between our genomes and our lived experiences.

## References

[1] Engel GL . The need for a new medical model: a challenge for biomedicine. Science. (1977) 196:129–36. doi: 10.1126/science.847460, PMID: 847460

[2] Monroe SM Simons AD . Diathesis-stress theories in the context of life stress research: implications for the depressive disorders. Psychol Bull. (1991) 110:406–25. doi: 10.1037/0033-2909.110.3.406, PMID: 1758917

[3] Sonuga-Barke EJS Kennedy M Kumsta R Knights N Golm D Rutter M . Child-to-adult neurodevelopmental and mental health trajectories after early life deprivation: the young adult follow-up of the longitudinal English and Romanian Adoptees study. Lancet. (2017) 389:1539–48. doi: 10.1016/S0140-6736(17)30045-4, PMID: 28237264

[4] Caspi A Sugden K Moffitt TE Taylor A Craig IW Harrington H . Influence of life stress on depression: moderation by a polymorphism in the 5-HTT gene. Science. (2003) 301:386–9. doi: 10.1126/science.1083968, PMID: 12869766

[5] Border R Johnson EC Evans LM Smolen A Berley N Sullivan PF . No support for historical candidate gene or candidate gene-by-interaction hypotheses for major depression across multiple large samples. Am J Psychiatry. (2019) 176:376–87. doi: 10.1176/appi.ajp.2018.18070881, PMID: 30845820 PMC6548317

[6] Lewis CM Vassos E . Polygenic risk scores: From research tools to clinical instruments. Genome Med. (2020) 12:44. doi: 10.1186/s13073-020-00742-5, PMID: 32423490 PMC7236300

[7] McIntosh AM Sullivan PF Lewis CM . Uncovering the genetic architecture of major depression. Neuron. (2019) 102:91–103. doi: 10.1016/j.neuron.2019.03.022, PMID: 30946830 PMC6482287

[8] Fang Y Scott L Song P Burmeister M Sen S . Genomic prediction of depression risk and resilience under stress. Nat Hum Behav. (2019) 4:111–8. doi: 10.1038/s41562-019-0759-3, PMID: 31659322 PMC6980948

[9] Peter HL Giglberger M Streit F Frank J Kreuzpointner L Rietschel M . Association of polygenic scores for depression and neuroticism with perceived stress in daily life during a long-lasting stress period. Genes Brain Behav. (2023) 22:e12872. doi: 10.1111/gbb.12872, PMID: 37876358 PMC10733580

[10] Nelemans SA Boks M Lin B Oldehinkel T Van Lier P Branje S . Polygenic risk for major depression interacts with parental criticism in predicting adolescent depressive symptom development. J Youth Adolescence. (2021) 50:159–76. doi: 10.1007/s10964-020-01353-4, PMID: 33230654 PMC7815554

[11] He Q Li JJ . A gene-environment interaction study of polygenic scores and maltreatment on childhood ADHD. Res Child Adolesc Psychopathol. (2022) 50:309–19. doi: 10.1007/s10802-021-00873-2, PMID: 34599701 PMC8891039

[12] Trotta A Iyegbe C Di Forti M Sham PC Campbell DD Cherny SS . Interplay between schizophrenia polygenic risk score and childhood adversity in first-presentation psychotic disorder: A pilot study. PLoS One. (2016) 11:e0163319. doi: 10.1371/journal.pone.0163319, PMID: 27648571 PMC5029892

[13] Cleary JL Fang Y Zahodne LB Bohnert ASB Burmeister M Sen S . Polygenic risk and social support in predicting depression under stress. Am J Psychiatry. (2023) 180:139–45. doi: 10.1176/appi.ajp.21111100, PMID: 36628515 PMC10355168

[14] Wang R Study LC Hartman CA Snieder H . Stress-related exposures amplify the effects of genetic susceptibility on depression and anxiety. Trans Psychiatry. (2023) 13:27. doi: 10.1038/s41398-023-02327-3, PMID: 36717542 PMC9886926

[15] Colodro-Conde L Couvy-Duchesne B Zhu G Coventry W Byrne E Gordon S . A direct test of the diathesis–stress model for depression. Mol Psychiatry. (2018) 23:1590–6. doi: 10.1038/mp.2017.130, PMID: 28696435 PMC5764823

[16] Agerbo E Trabjerg BB Børglum AD Schork AJ Vilhjálmsson BJ Pedersen CB . Risk of early-onset depression associated with polygenic liability, parental psychiatric history, and socioeconomic status. JAMA Psychiatry. (2021) 78:387. doi: 10.1001/jamapsychiatry.2020.4172, PMID: 33439215 PMC7807393

[17] Musliner KL Andersen KK Agerbo E Albiñana C Vilhjalmsson BJ Rajagopal VM . Polygenic liability, stressful life events and risk for secondary-treated depression in early life: A nationwide register-based case-cohort study. psychol Med. (2023) 53:217–26. doi: 10.1017/S0033291721001410, PMID: 33949298

[18] Østergaard SD Trabjerg BB Als TD Climent CA Privé F Vilhjálmsson BJ . Polygenic risk score, psychosocial environment and the risk of attention-deficit/hyperactivity disorder. Trans Psychiatry. (2020) 10:335. doi: 10.1038/s41398-020-01019-6, PMID: 33009369 PMC7532146

[19] Mullins N Power RA Fisher HL Hanscombe KB Euesden J Iniesta R . Polygenic interactions with environmental adversity in the aetiology of major depressive disorder. psychol Med. (2016) 46:759–70. doi: 10.1017/S0033291715002172, PMID: 26526099 PMC4754832

[20] Coleman JRI Peyrot WJ Purves KL Davis KAS Rayner C Choi SW . Genome-wide gene-environment analyses of major depressive disorder and reported lifetime traumatic experiences in UK Biobank. Mol Psychiatry. (2020) 25:1430–46. doi: 10.1038/s41380-019-0546-6, PMID: 31969693 PMC7305950

[21] Arnau-Soler A Adams MJ Generation Scotland, Major Depressive Disorder Working Group of the Psychiatric Genomics Consortium Hayward C Thomson PA . Genome-wide interaction study of a proxy for stress-sensitivity and its prediction of major depressive disorder. PLoS One. (2018) 13:e0209160. doi: 10.1371/journal.pone.0209160, PMID: 30571770 PMC6301766

[22] Suppli NP Andersen KK Agerbo E Rajagopal VM Appadurai V Coleman JRI . Genome-wide by environment interaction study of stressful life events and hospital-treated depression in the iPSYCH2012 sample. Biol Psychiatry Global Open Sci. (2022) 2:400–10. doi: 10.1016/j.bpsgos.2021.11.003, PMID: 36324662 PMC9616262

[23] Wannemüller A Kumsta R Jöhren H-P Eley TC Teismann T Moser D . Genes in treatment: Polygenic risk scores for different psychopathologies, neuroticism, educational attainment and IQ and the outcome of two different exposure-based fear treatments. World J Biol Psychiatry. (2021) 22:699–712. doi: 10.1080/15622975.2021.1907708, PMID: 33970774

[24] Rayner C Coleman JRI Purves KL Hodsoll J Goldsmith K Alpers GW . A genome-wide association meta-analysis of prognostic outcomes following cognitive behavioural therapy in individuals with anxiety and depressive disorders. Trans Psychiatry. (2019) 9:150. doi: 10.1038/s41398-019-0481-y, PMID: 31123309 PMC6533285

[25] Arnau-Soler A Macdonald-Dunlop E Adams MJ Clarke T-K MacIntyre DJ Milburn K . Genome-wide by environment interaction studies of depressive symptoms and psychosocial stress in UK Biobank and Generation Scotland. Trans Psychiatry. (2019) 9:14. doi: 10.1038/s41398-018-0360-y, PMID: 30718454 PMC6361928

[26] Eley TC Hudson JL Creswell C Tropeano M Lester KJ Cooper P . Therapygenetics: The 5HTTLPR and response to psychological therapy. Mol Psychiatry. (2012) 17:236–7. doi: 10.1038/mp.2011.132, PMID: 22024766 PMC3272476

[27] Gibson G . On the utilization of polygenic risk scores for therapeutic targeting. PLoS Genet. (2019) 15:e1008060. doi: 10.1371/journal.pgen.1008060, PMID: 31022172 PMC6483161

[28] Belsky J Pluess M . Beyond diathesis stress: differential susceptibility to environmental influences. Psychol Bull. (2009) 135:885–908. doi: 10.1037/a0017376, PMID: 19883141

[29] Pluess M Belsky J . Vantage sensitivity: individual differences in response to positive experiences. Psychol Bull. (2013) 139:901–16. doi: 10.1037/a0030196, PMID: 23025924

[30] Peyrot WJ Van der Auwera S Milaneschi Y Dolan CV Madden PAF Sullivan PF . Does Childhood Trauma Moderate Polygenic Risk for Depression? A Meta-analysis of 5765 Subjects From the Psychiatric Genomics Consortium. Biological Psychiatry. (2018) 84:138–147. doi: 10.1016/j.biopsych.2017.09.009, PMID: 29129318 PMC5862738

[31] Hyde CL Nagle MW Tian C Chen X Paciga SA Wendland JR . Identification of 15 genetic loci associated with risk of major depression in individuals of European descent. Nature Genetics. (2016) 48:1031–1036., PMID: 27479909 10.1038/ng.3623 PMC5706769

[32] Demontis D Walters RK Martin J Mattheisen M Als TD Agerbo E Baldursson G . Discovery of the first genome-wide significant risk loci for attention deficit/hyperactivity disorder. Nature Genetics. (2019) 51:63–75. doi: 10.1038/s41588-018-0269-7, PMID: 30478444 PMC6481311

[33] Purves KL Coleman JRI Meier SM Rayner C Davis KAS Cheesman R . A major role for common genetic variation in anxiety disorders. Molecular Psychiatry. (2020) 25:3292–3303. doi: 10.1038/s41380-019-0559-1, PMID: 31748690 PMC7237282

